# Efficacy of Optically Pumped Magnetometers in Detecting Activity From the Cerebellar Cortex

**DOI:** 10.1002/hbm.70514

**Published:** 2026-03-26

**Authors:** Santtu Roos, Matti Hämäläinen, Joonas Iivanainen

**Affiliations:** ^1^ Department of Neuroscience and Biomedical Engineering Aalto University Espoo Finland

**Keywords:** cerebellum, forward modeling, magnetoencephalography, optically pumped magnetometers

## Abstract

The cerebral cortex has been extensively studied using magnetoencephalography (MEG), but the cerebellum has received less attention, partly due to technical limitations. Recent advances in high‐resolution anatomical modeling enable surface‐based analysis of cerebellar activity. At the same time, MEG technology has evolved, with on‐scalp systems employing optically pumped magnetometers (OPMs) emerging as an alternative for conventional superconducting quantum interference device (SQUID)‐based systems. In contrast to rigid one‐size‐fits‐all SQUID sensor helmets, OPMs allow flexible positioning of the sensors on the participant's scalp to provide improved coverage of the cerebellum. To assess the benefits provided by OPMs in detecting cerebellar activity, we conducted simulations using a high‐resolution model of the human cerebellum, where we compared OPM arrays consisting either of single‐axis or triaxial sensors to commercial SQUID sensor arrays. We show that both OPM types measure stronger net signals from across the cerebellum compared to the SQUID‐based systems. OPMs also reduce signal correlations between the cerebral and cerebellar cortices, improving source separability. Increasing the number of OPM sensors leads to larger gains in total information capacity compared to SQUIDs. In all metrics, triaxial OPMs outperformed single‐axis configurations. These results suggest that already a 102‐sensor, triaxial OPM‐based on‐scalp MEG system could substantially improve noninvasive electrophysiological studies of the human cerebellum.

## Key Points

- We studied the use of OPMs over conventional SQUID sensors in detecting cerebellar activity.
- Our results indicate clear benefits for OPMs in terms of sensitivity, lead‐field correlations between the cortex and the cerebellum, as well as total information.
- Importantly, our results indicate that the improvements provided by OPMs in measuring the cerebellum can already be achieved with 102 triaxial OPM sensors covering the whole head.

## Graphical abstract

We studied the utility of OPMs and conventional SQUID sensors in detecting cerebellar activity. Our results indicate clear benefits for OPMs in terms of sensitivity, lead‐field correlations between the cortex and the cerebellum, and total information, with these improvements already achievable with 102 triaxial OPM sensors covering the whole head.

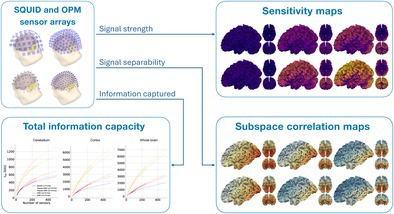

## 1 Introduction

The cerebellum has traditionally been associated with lower‐level brain functions such as coordination and fine‐tuning of movements. However, an increasing number of studies now highlight its involvement in higher‐order cognitive processes, including language and working memory (Guell et al. 2018; Koziol et al. 2014; Stoodley and Schmahmann 2009), implicit memory functions such as motor learning and internal modeling (Christian and Thompson 2003; Hadjiosif et al. 2024; Thach 1997), as well as reinforcement learning (Huvermann et al. 2025). The cerebellum has also been implicated in several neurological and psychiatric conditions, including schizophrenia (Andreasen and Pierson 2008; Picard et al. 2008), essential tremor (Cerasa and Quattrone 2016; Choe et al. 2016; Grimaldi and Manto 2013; Schnitzler et al. 2009), and Parkinson's disease (Ma et al. 2007; Wu and Hallett 2013; Wu et al. 2009; Yu et al. 2007). Notably, the cerebellum contains over 70% of the brain's neurons and maintains extensive reciprocal connections with both subcortical and cortical regions (Andersen et al. 1992; Herculano‐Houzel 2009). The cerebellar cortex has expanded significantly in humans compared to non‐human primates, pointing toward involvement in uniquely human traits such as language (Sereno et al. 2020).

Despite a well‐established anatomical understanding, the cerebellum remains relatively understudied in terms of its electrophysiological contributions to motor and cognitive functions (Schmahmann and Sherman 1998; Wu and Hallett 2013). This gap is not due to a lack of relevance, but rather the limitations of available neuroimaging tools. Although PET and fMRI have revealed important aspects of cerebellar metabolism and hemodynamics (Stoodley and Schmahmann 2009), their indirect nature and limited temporal resolution combined with the cerebellum's tightly folded anatomy have constrained efforts to study its neural activity in detail.

Magnetoencephalography (MEG) is a non‐invasive neuroimaging technique that measures the tiny magnetic fields generated by neural currents with a millisecond resolution (Hämäläinen et al. 1993). These weak fields were first recorded by David Cohen in the 1960s (Cohen 1968), and MEG has since found applications in both basic neuroscience (see, e.g., Baillet 2017) and clinical contexts, including epilepsy (Stefan and Trinka 2017), dementia (López‐Sanz et al. 2018), traumatic brain injury (Huang et al. 2014), and autism (Roberts et al. 2019).

As discussed in a recent review by Andersen et al. (2020), MEG can detect cerebellar activity, but challenges remain. First, the cerebellum's deep location within the skull increases the source‐to‐sensor distance, and its highly folded structure leads to field cancellation from oppositely oriented dipoles (Ahlfors et al. 2010). Second, the lack of high‐resolution individual surface models of the cerebellum due to insufficient spatial resolution of conventional MRI scanners has limited the accuracy of source estimation. Third, current MEG systems based on superconducting quantum interference device (SQUID) sensors are suboptimal to measure cerebellar activity. SQUIDs are highly sensitive ($3\;\text{fT}/\sqrt{\mathrm{Hz}}$), but they require cryogenic cooling, which imposes an insulating layer between the scalp and the sensors. This insulating layer increases the sensor‐to‐scalp distance to more than 2 cm, which leads to a difficulty in detecting far away activity and loss of spatial detail in the measurements. This arrangement also fixes the geometry of the sensor array, likely resulting in a suboptimal array for cerebellar measurements, limiting the ability to detect cerebellar signals with SQUID‐based MEG. Recently, however, there have been significant advances in overcoming these obstacles.

Samuelsson et al. proposed a method to obtain a high‐resolution surface model of an individual's cerebellum (Samuelsson, Rosen, et al. 2020). They morphed to the individual's head model the high‐resolution cerebellum model that was obtained by Sereno and colleagues by scanning a human cerebellum specimen with a 9.4 T ex vivo MRI (Sereno et al. 2020). Using this model and established forward modeling techniques (Hämäläinen et al. 1993; Hämäläinen and Sarvas 1989; Mosher et al. 1995), they further simulated cerebellar activity and assessed its detectability using SQUID‐based MEG systems (Samuelsson, Sundaram, et al. 2020). They found that measuring SQUID‐MEG from the cerebellum is possible, resulting in signal amplitudes that are only 30%–60% weaker than those from cortical sources.

The limitations of SQUID‐based systems have spurred growing interest to use optically pumped magnetometers (OPMs) (Budker and Romalis 2007; Kimball et al. 2013) for MEG. OPMs are compact sensors that operate at near room temperature and do not require cryogenic cooling, allowing them to be flexibly positioned directly on the scalp with a distance of about 5 mm between the sensor's sensitive element and the scalp. Although their typical sensitivity (around $10–20\;\text{fT}/\sqrt{\mathrm{Hz}}$) is somewhat worse than that of SQUIDs, their proximity to the brain and flexibility in sensor placement provide substantial advantages over rigid SQUID‐based sensor arrays (Iivanainen et al. 2017). Typically, OPM systems use single‐ or dual‐axis sensors, but triaxial OPMs (Beato et al. 2018; Boto et al. 2022), which measure magnetic field along three orthogonal directions, are attracting increasing interest. Triaxial sensor arrays improve separation of neural signals from external interference (Brookes et al. 2021; Nurminen et al. 2013), enhance SNR, and reduce motion‐related artefacts (Rea et al. 2022). They also provide more uniform cortical coverage, particularly, in infants and children (Boto et al. 2022; Iivanainen et al. 2017). The OPM sensors have been used to measure signals from brain areas that are not covered well by SQUID‐based MEG, such as the hippocampus (Barry et al. 2019; Tierney et al. 2021) and the cerebellum (Lin et al. 2019). However, the benefits of OPM‐based MEG in measuring the human cerebellum over SQUID‐MEG have not been systematically quantified.

In this paper, we investigate the performance of OPM‐MEG sensor arrays in measuring activity from the human cerebellum cortex using the high‐resolution model of the human cerebellum with over 4 million vertices, as proposed by Samuelsson et al. (2020). We compare single‐axis and triaxial OPM arrays to commercial SQUID sensor arrays using three complementary forward‐model‐based metrics. First, we compute sensitivity maps to assess signal strength and spatial coverage across the cerebellum. Second, we use subspace angle analysis to evaluate how well each system disambiguates cerebellar signals from cortical sources. Third, we estimate the total information capacities of the arrays. Our analyses reveal which cerebellar regions are most accessible and which sensor configurations are most effective, guiding future study design and improving the reliability of non‐invasive cerebellar MEG.

## 2 Methods

### 2.1 Anatomical Models

To model the cerebellum in our simulations, we used a high‐resolution geometric model developed by Sereno et al. (2020), based on an ex vivo human brain scanned post‐mortem with a 9.4‐T MRI system. The dataset includes T2*‐weighted and 3D FLASH images from a 62‐year‐old previously healthy female, acquired at an isotropic voxel resolution of 0.19 × 0.19 × 0.19 mm^3^. The volume was manually refined to correct topological errors.

The detailed cerebellum model was morphed to match the anatomy of the MNE sample subject using the CereMegBellum (CMB) package (Samuelsson, Rosen, et al. 2020). MRI data for this subject were acquired with a Siemens 1.5T Sonata scanner using an MPRAGE sequence. Cortical surfaces and the subject's cerebellar outline were reconstructed with FreeSurfer (Fischl 2012), after which the ex vivo surface was aligned to the individual anatomy using the ARCUS method (Samuelsson, Rosen, et al. 2020).

We constructed source spaces on the cortical and cerebellar surfaces in Python 3.8 using the MNE‐Python software (v1.6.1) (Gramfort et al. 2013; Larson 2024). Primary‐current distribution was discretized to dipoles oriented normal to the local cortical or cerebellar surface. For the cerebral cortex, the source space consisted of 169,495 vertices. The cerebellum was modeled similarly, with a high‐resolution source space containing 4,573,612 vertices. We also generated a downsampled version of the cerebellar source space with 99,856 vertices to reduce the computational complexity in simulations that involve calculation of pairwise source distances. From here on, we refer to the cerebral cortex as “cortex” and the cerebellar cortex as “cerebellum.” Depending on the simulation, we used either a cortical or a cerebellar source space or their combination.

### 2.2 Sensor Arrays

MEG forward simulations were performed for four sensor types: SQUID magnetometers of the MEGIN (formerly Elekta Neuromag) Vectorview system (Espoo, Finland), SQUID axial gradiometers of the CTF MEG Neuro Innovations Inc. system (Coquitlam, Canada), and single‐ and tri‐axis OPM sensors with their geometry modeled according to Gen‐1 OPM by QuSpin Inc. (Louisville, USA). All sensor types were modeled as in the MNE‐python software.

We formed six sensor arrays (SQUID 102, OPM 102, Triaxial OPM 102, CTF, OPM 275, and Triaxial OPM 275) as follows. The SQUID 102 sensor array was obtained directly from the MNE's sample dataset; it presents how the sensor array was in relation to the subject's head during the measurement. The CTF SQUID system with 275 axial gradiometers with a baseline of 50 mm was modeled by manually placing the MNE sample subject inside a CTF helmet. The smallest sensor‐to‐scalp distance in SQUID 275 is approximately 22 mm, which is reasonable for real‐world measurements and closely matches the SQUID 102 minimum scalp‐to‐sensor distance of 24 mm used in our simulations.

**FIGURE 1:**
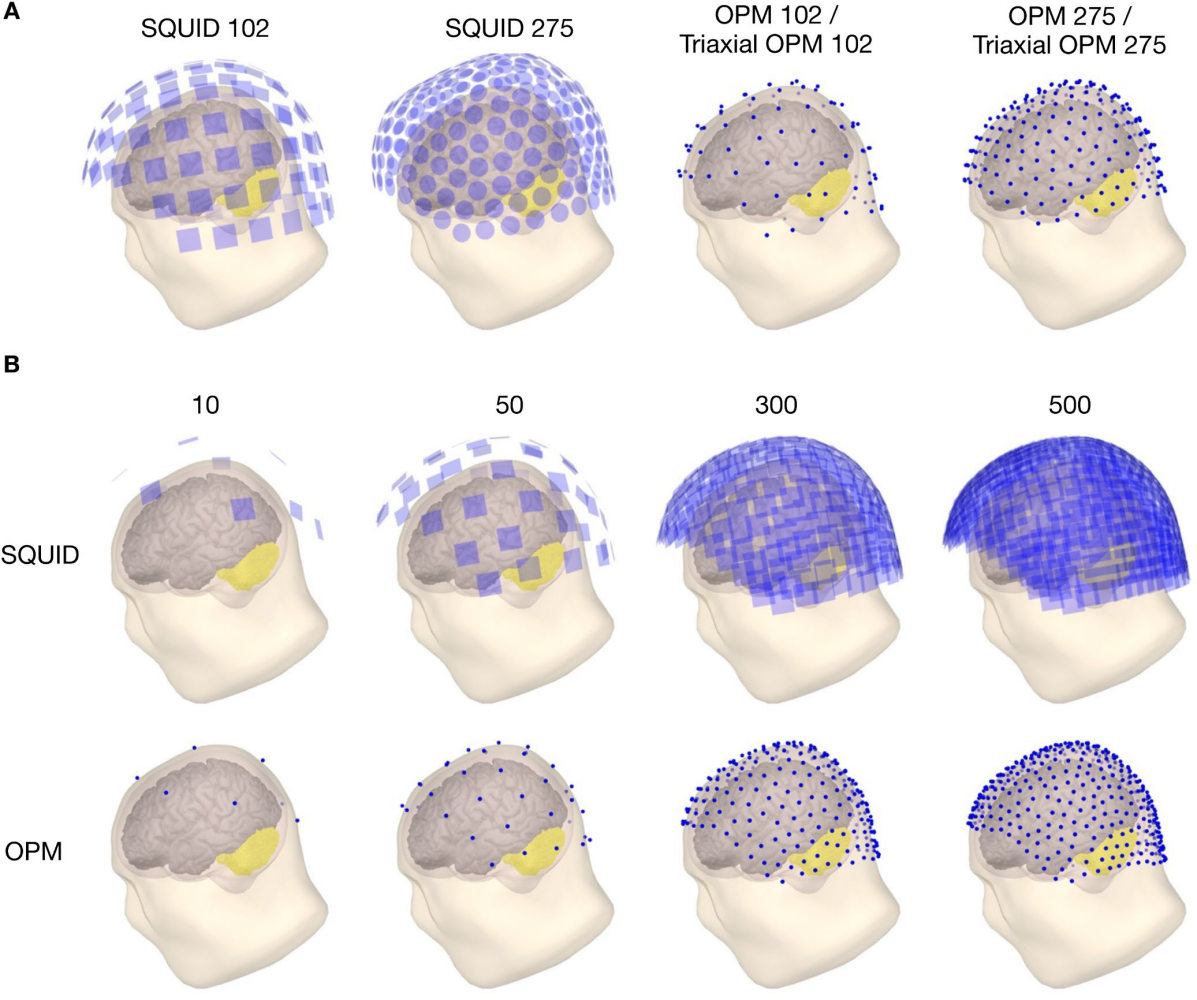
(A) Sensor arrays for SQUID 102, SQUID 275, OPM 102, and OPM 275. Triaxial OPM arrays follow the same layouts as the corresponding single‐axis OPM arrays, but with three orthogonal channels in one sensor at each location. Thus, for example, Triaxial OPM 102 contains 306 channels, while OPM 102 contains 102 channels. All arrays use magnetometers, except SQUID 275, which uses axial gradiometers with a 50 mm baseline. (B) Examples of generated uniform SQUID (top row) and OPM (bottom row) arrays of 10, 50, 300, and 500 sensors that were used in the total information capacity calculations. The cerebellar source space is highlighted in yellow in all figures.

We constructed OPM sensor arrays from the SQUID 102 and CTF SQUID sensor positions by projecting them onto the scalp so that the distances between the OPMs and the scalp were 5 mm. The orientation of each OPM sensor was set to follow the local scalp surface normal (“radial” orientation). For triaxial OPM arrays, each triaxial OPM sensor comprised three channels measuring the radial and the two tangential components of the magnetic field. This resulted in a total of four OPM sensor arrays: 102‐sensor single and triaxial OPM arrays obtained from the SQUID 102 array and 275‐sensor single and triaxial OPM arrays obtained from the SQUID 275 array. The resulting sensor arrays are shown in Figure 1A.

To analyze how total information capacity changes as a function of the number of sensors, we generated custom OPM and SQUID (Vectorview magnetometer) sensor layouts with varying numbers of sensors. To do this, we adapted the uniform spatial sampling strategy proposed by Iivanainen et al. (2021), which places the sensors so that they uniformly cover the desired region. Here, we briefly outline how we used that method.

We started by constructing a dense mesh of candidate sensor positions on the scalp surface. For the SQUID array, we created the mesh from its sensor placements using Delaunay triangulation and then subdivided it, resulting in a total of 5821 vertices. For the OPM arrays, we first projected the sensors closer to the scalp, as described above, and then generated a mesh with the same procedure and number of vertices. This ensured that OPM and SQUID arrays had comparable candidate sensor placements. A Laplace basis was computed over this surface using the Dirichlet boundary condition (Jacobson 2024). These functions were used to generate a covariance matrix with uniform variance on each function. We then applied the farthest‐point sampling algorithm (Eldar et al. 1997; Schlömer et al. 2011) in this space to yield sensor positions that have approximately uniform spatial distribution on the surface (Iivanainen et al. 2021). Layouts were generated for a range of sensor counts from 10 to 500. These served as the basis for later forward model and information capacity computations. Examples of these sensor arrays are shown in Figure 1B.

### 2.3 Forward Models

We used a three‐compartment, piecewise homogeneous boundary element method (BEM) model of the head (Mosher et al. 1995), with conductivities of 0.3 S/m for brain and scalp, and 0.006 S/m for skull as defaulted in MNE. BEM computations were performed in MNE‐Python using MRI data from the MNE sample dataset. All three BEM surfaces were modeled with 2562 vertices each. To minimize boundary‐related errors, source vertices within 3 mm of the inner skull were excluded. This constraint resulted in source spaces comprising 166,833 vertices for the cortex, 4,160,046 vertices for the high‐resolution cerebellum, and 91,373 vertices for its downsampled version. In the forward calculations, the output of the different types of sensors was computed using the numerical integration approximations implemented in the MNE‐Python software (see Gramfort et al. 2014).

### 2.4 Simulation Metrics

#### 2.4.1 Sensitivity Maps

To compare the sensor arrays in detecting cerebellar activity, we calculate their sensitivity, which measures the signal size originating from a dipole source in the cerebellum. We also calculate the sensitivity to cerebral sources to facilitate comparison of signal strength between the cortex and the cerebellum.

The relationship between MEG signals and source activity is given by:

(1) b=Lj,

where $b\in {\mathbb{R}}^{m}$ represents the measurements collected by m sensors, $L\in {\mathbb{R}}^{m\times n}$ is the lead‐field matrix, and $j\in {\mathbb{R}}^{n}$ denotes the n source amplitudes. The rows of the lead‐field matrix are the sensor lead fields, that is, the sensitivity patterns of the sensors to the sources, while the columns are the field patterns, or topographies, of the sources. The sensor array's sensitivity $s_{j}$ to a source location can be quantified by the Euclidean norm of the topography of the source:

(2) $$s_{j}={\left\|L_{:,j}\right\|}_{2},$$

where $L_{:,j}$ denotes the j‐th column of L. This norm captures the strength of the magnetic field generated by the dipole at that location, as measured by the sensor array. By calculating the sensitivity of each source, we obtain the sensitivity map. For the sensitivity calculations, we set the dipole moment of the source to 100 nAm, similar to Samuelsson et al. (2020).

#### 2.4.2 Correlation Maps

To assess the ability of different sensor arrays to separate signals originating from the cerebellum and the cortex, we adopted a subspace angle‐based method. Subspace (or principal) angles describe the angular relationship between two subspaces and can be used to quantify how distinguishable the corresponding MEG topographies from different brain regions are. This kind of analysis has been previously applied in MEG and EEG studies (Krishnaswamy et al. 2017; Mosher 2000). The following introduction to the method follows the algorithm presented by Björck and Golub (1973).

Let $L_{A}\in {\mathbb{R}}^{m\times q}$ and $L_{B}\in {\mathbb{R}}^{m\times p}$ be lead‐field matrices of the same sensor array for two source regions, where m is the number of sensors and q and p are the number of sources in each region. We compute their singular value decompositions $L_{A}=U_{A}S_{A}V_{A}^{⊤}$ and $L_{B}=U_{B}S_{B}V_{B}^{⊤}$, where $U_{A}\in {\mathbb{R}}^{m\times \mathrm{rank}\left(A\right)}$ and $U_{B}\in {\mathbb{R}}^{m\times \mathrm{rank}\left(B\right)}$. Then we form $C=U_{A}^{⊤}U_{B}$ and compute its singular value decomposition $C=U_{C}S_{C}V_{C}^{⊤}$. It can be shown that the diagonal elements of $S_{C}$ are the cosines of the subspace angles $\theta_{1},\ldots ,\theta_{r}$ between $\text{range}\left(L_{A}\right)$ and $\text{range}\left(L_{B}\right)$, with $r=\min\left(\mathrm{rank}\left(A\right),\mathrm{rank}\left(B\right)\right)$. The cosines of the principal angles between the two subspaces are equivalent to the canonical correlations between the sets of vectors spanning these subspaces, providing a measure of how aligned or correlated the subspaces are. Since these subspaces are generally multidimensional, the result is not a single angle, but a set of principal angles $\theta_{1},\ldots ,\theta_{r}$.

We computed subspace angles between the cortex and the cerebellum. Unlike the sensitivity and total information measures, this analysis was performed on source patches rather than individual dipoles, because subspace angles quantify the alignment between multidimensional subspaces, and correlations between a single cortical dipole and the entire cerebellum are not meaningful in this context. On the cortex, local patches were defined as sets of vertices within a geodesic distance of 10 mm from a given seed vertex, and the corresponding lead‐field matrix of each patch was extracted. Geodesic distances were obtained using Dijkstra's algorithm on the cortical mesh as implemented in MNE. The chosen 10 mm radius patch size corresponds to the 100 nAm dipole used in the sensitivity map calculations (Samuelsson, Sundaram, et al. 2020).

We then computed subspace angles between cortical patches and the cerebellum using two approaches. In the first, a cortical patch of 10 mm radius was fixed and compared to cerebellar patches of equal size. This choice is motivated by the findings of Murakami and Okada (2015), who found that synchronously active neuronal populations exhibit comparable current dipole moment densities of about $1\;\mathrm{nAm}/{\mathrm{mm}}^{2}$ in the neocortex, hippocampus, and cerebellar cortex. Therefore, using equal patch size in the cortex and the cerebellum leads to the same current dipole moment in both. For each cerebellar patch, the cosines of the subspace angles were averaged to yield a single correlation value, which was then assigned to that cerebellar patch. The procedure was repeated across the cerebellum with overlapping patches placed at 15 mm seed spacing, producing a cerebellar correlation map with respect to the chosen cortical patch. This approach highlights how subspace‐angle correlations distribute across the cerebellum for a fixed cortical patch.

In the second analysis, each cortical 10 mm radius patch was compared to the full cerebellar lead field rather than to individual cerebellar patches. For a given cortical patch, the cosines of the subspace angles between that cortical patch and the cerebellum were averaged to yield one correlation value, which was assigned to the cortical patch. This procedure was repeated across the cortex with 15 mm seed spacing; the spatially overlapping values from nearby patches were averaged. The result was a full cortical correlation map with respect to the whole cerebellum. By averaging over the full cerebellum, the second approach emphasizes the spatial organization across cortical regions while avoiding the computational cost of patch‐to‐patch comparisons.

In addition to averaging the correlation values between the cortical patch and the cerebellum, we also quantified the percentage of highly correlated subspaces (correlation > 0.5) between them. This produces a map over the cortex that shows the spatial distribution of the number of highly correlated subspaces between the cerebellum and the corresponding location of the cortex.

#### 2.4.3 Total Information Capacity

To evaluate the performance of different MEG sensor arrays, we computed their total information capacity $I_{\mathrm{tot}}$ (Iivanainen et al. 2017; Kemppainen and Ilmoniemi 1989; Schneiderman 2014). This metric quantifies the amount of information conveyed by the sensor array and takes into account the sensor configuration, the sensor noise level, and the overlap in the sensor lead fields.

We briefly describe how the total information capacity is calculated; for a thorough description see, e.g., (Iivanainen et al. 2017). We first whiten the sensor lead fields using a homoscedastic noise covariance matrix with sensor noise variance on its diagonal elements. The whitened lead fields are then orthogonalized via eigenvalue decomposition to avoid overestimating information due to correlated sensor lead fields. The total information capacity is then calculated by summing the Shannon information over the orthogonalized channels:

$$I_{\mathrm{tot}}=\frac{1}{2}\sum_{i=1}^{N_{c}}\log_{2}\left(\mathrm{SN}R_{i}^{\prime }+1\right),$$

where $\mathrm{SN}R_{i}^{\prime }$ are the signal‐to‐noise ratios of the orthogonalized channels, and $N_{c}$ is the number of orthogonalized channels that is equal to the number of total channels in the array.

We calculate the total information for the sensor arrays presented in Figure 1A and as well as for uniform SQUID and OPM arrays with varying numbers of sensors shown in Figure 1B. The OPMs were assumed to have a noise level of 10 or $15\;\text{fT}/\sqrt{\mathrm{Hz}}$, while the SQUID magnetometers and axial gradiometers were both assigned a noise level of $3\;\text{fT}/\sqrt{\mathrm{Hz}}$, reflecting typical performance under comparable conditions. We calculated $I_{\mathrm{tot}}$ separately for the cerebellum (downsampled version with 91,373 vertices) and the cortex, as well as for their combined source space, using a grid spacing of 2 mm. The source variance $q^{2}$ was chosen so that, in the whole‐brain calculation with the 500‐sensor SQUID array, the average SNR across sources equaled 1 ($q^{2}$ has been set similarly also in Iivanainen et al. 2017). This value was applied to all sensor types and arrays, in all three source‐space cases.

## 3 Results

### 3.1 Sensitivity Maps

**FIGURE 2:**
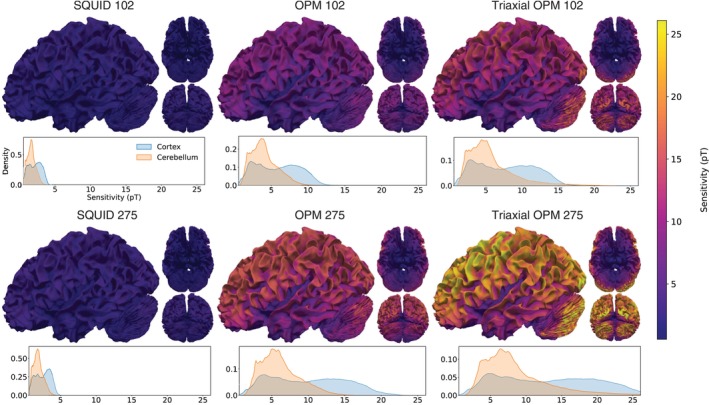
Sensitivity maps for SQUID 102, OPM 102, Triaxial OPM 102, SQUID 275, OPM 275, and Triaxial OPM 275 sensor arrays. Each map is scaled between its 1st and 99th percentile of the sensitivity; the colorbar is therefore scaled between the 1st and 99th percentiles of all sensitivity maps. The maps are shown from lateral, posterior, and inferior views. Below each sensitivity map, a density plot illustrates the distribution of both cortical (blue) and cerebellar (red) sensitivity values within the colorbar range.

Sensitivity maps for the different sensor arrays are shown in Figure 2, with accompanying density plots illustrating the distribution of sensitivity values. The overall spatial distribution of sensitivity is similar across the sensor arrays. Sensitivity is highest near the skull and in the posterior lobe of the cerebellum, particularly, between the primary and horizontal fissures. Lower sensitivity is observed in medial and lateral regions, including the vermal area, and the lowest values occur in deep cerebellar structures, especially near the cerebellar peduncles and tonsil.

For SQUID 102 and 275, most sensitivity values remain below 3 pT for the cerebellum, with 95th percentiles of 2.7 and 3.1 pT, respectively. The difference between the two is modest: SQUID 275 achieves slightly higher maximum sensitivity, but their main density peaks remain close (1.6 pT for SQUID 102 and 1.8 pT for SQUID 275). OPM 102 reaches a 95th percentile of 7.4 pT for the cerebellum, while OPM 275 yields even higher signals with a 95th percentile of 11.1 pT. The peak density for OPM 102 is around 3.7 pT, whereas OPM 275 shows a broader and higher peak at 5.1 pT. Triaxial OPM 102 extends the 95th percentile sensitivity range up to about 12.5 pT, and Triaxial OPM 275 reaches 18.6 pT. Compared to OPM 102, Triaxial OPM 102 broadens and shifts the main cerebellar density peak from 3.7 to 4.4 pT, while Triaxial OPM 275 shifts it further to around 6.3 pT.

When averaged over all cerebellar sources, the OPM arrays increase sensitivity to the cerebellum relative to SQUID 102 by factors of 2.4, 3.7, 3.4, and 5.2 for OPM 102, OPM 275, Triaxial OPM 102, and Triaxial OPM 275, respectively. The same ratio of SQUID 275 to SQUID 102 is 1.1.

When comparing average sensitivities, cortical values exceeded cerebellar values for all arrays, with cortex‐to‐cerebellum ratios of 1.30 (SQUID 102), 1.36 (SQUID 275), 1.46 (OPM 102), 1.64 (OPM 275), 1.35 (Triaxial OPM 102), and 1.53 (Triaxial OPM 275). Thus, while OPM arrays substantially increased absolute sensitivity for both regions (average sensitivity rising from 1.67 pT to 8.69 pT in the cerebellum and from 2.17 to 13.31 pT in the cortex), they did not narrow the cortex–cerebellum sensitivity gap.

**FIGURE 3:**
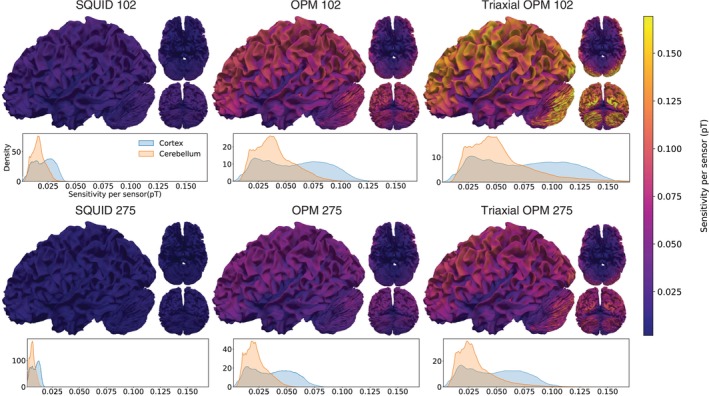
Sensitivity maps normalized by the number of sensors for SQUID 102, OPM 102, Triaxial OPM 102, SQUID 275, OPM 275, and Triaxial OPM 275 sensor arrays. Each map is scaled between its 1st and 99th percentile of sensitivity. The maps are shown from lateral, posterior, and inferior views. Below each sensitivity map, a density plot illustrates the distribution of the sensitivity values within the colorbar range.

Figure 3 presents the sensitivity maps of Figure 2, but with the sensitivity normalized by the number of sensors in each array. This normalization highlights higher per‐sensor sensitivity in the 102‐sensor arrays (top row) compared to the 275‐sensor arrays (bottom row). The highest per‐sensor sensitivity is observed with Triaxial OPM 102 configuration, while the lowest is seen with SQUID 275 array. The results indicate sub‐linear gain in sensitivity as a function of the sensor count.

### 3.2 Correlation Maps

**FIGURE 4:**
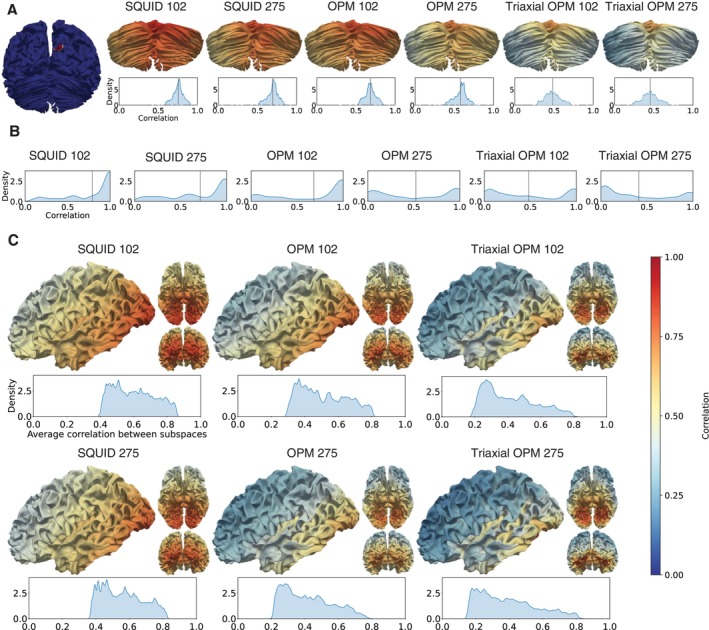
Subspace‐correlation analysis between the cortex and the cerebellum. (A) An example cortical seed patch (radius 10 mm) in the occipital lobe. The cerebellum was scanned with patches of equal size (seed spacing 15 mm), and the subspace correlations between the lead‐field matrices from the patches were averaged. Colormaps show the resulting average cerebellar correlations with the seed patch. Density plots below each map display the distributions of the average correlation, with their averages marked by dashed lines. (B) The correlations across all the lead‐field subspaces between the cortical patch shown in (A) and the whole cerebellum. Density plots show the distributions with averages marked by dashed lines. (C) The average subspace correlation between the cortical patch and the whole cerebellum as a function of the patch location on the cortex. Each cortical patch was compared with the full cerebellar lead field as in (B).

To illustrate the subspace angle‐based method, Figure 4A shows an example cortical patch in the occipital lobe and its average subspace correlation over similar patches in the cerebellum for the different sensor arrays. We generated these maps by scanning the cerebellum with patches of the same size as the cortical seed (radius 10 mm, seed spacing 15 mm). SQUID arrays yield the highest correlations, with average correlation (±standard deviation [SD]) of 0.75 (±0.06) and 0.69 (±0.07) for SQUID 102 and 275, respectively. OPM 102 lowers the average to 0.68 (±0.07), while OPM 275 reduces it further to 0.58 (±0.08). The lowest correlation values are obtained with triaxial OPM arrays: the averages for Triaxial OPM 102 and 275 are 0.48 (±0.10) and 0.45 (±0.10), respectively.

We then calculated full cortical correlation maps by comparing each cortical patch lead field to the full cerebellar lead field. Figure 4B shows the results for the same occipital patch as in Figure 4A. This approach yields higher correlations overall and produces different density distributions. However, when averaged, the results converge toward those in Figure 4A.

Figure 4C shows the full cortical correlation maps for all sensor arrays, together with density plots. SQUID 102 yields the highest correlations, with an average (±SD) of 0.60 (±0.12) and values ranging between 0.43 and 0.81 (the 5th and 95th percentiles, respectively). SQUID 275 performs slightly better than SQUID 102, averaging 0.56 (±0.12) with a 5th–95th percentile range of 0.39–0.77. OPM 102 lowers the mean correlation to 0.51 (±0.14), with a 5th–95th percentile range of 0.33–0.76, while Triaxial OPM 102 further reduces correlations to 0.42 (±0.16) within the range 0.22–0.71. OPM 275 lowers the mean to 0.42 (±0.14) with a 5th–95th percentile range of 0.23–0.68, and Triaxial OPM 275 reaches the lowest correlations, with an average of 0.39 (±0.17) and percentiles between 0.17 and 0.72. Across all arrays, correlations are highest in the occipital cortex, followed by temporal, parietal, and frontal regions. SQUID 102 and 275, and OPM 102 are the only arrays in which the average correlation exceeds 0.5.

**FIGURE 5:**
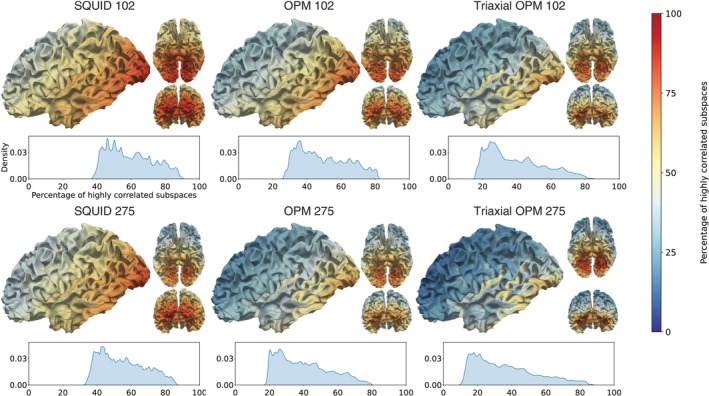
Percentage of highly correlated subspaces (r>0.5) between the cortical patches and the cerebellum for each sensor array. Values were derived from the full cortical–cerebellar correlation results as shown in Figure 4B. Density plots show the distributions of correlation values across cortical patches.

Figure 5 summarizes these results by showing the percentage of highly correlated subspaces (r>0.5). The trends match the full cortical maps: SQUID 102 retains the largest proportion of highly correlated subspaces, whereas Triaxial OPM 275 shows the smallest.

### 3.3 Information Capacity

Total information capacities ($I_{\mathrm{tot}}$, bits) for the cerebellum, cortex, and whole brain are summarized in Table 1 for the sensor arrays used in the previous simulations. Here, the source variance $q^{2}$ was scaled so that the average SNR across the sources in the whole brain equaled 1 for SQUID 102. SQUID noise was fixed at $3\;\text{fT}/\sqrt{\mathrm{Hz}}$, and OPM arrays were evaluated at 10 and $15\;\text{fT}/\sqrt{\mathrm{Hz}}$ noise levels. Since the effects arising from sensor overlap were not modeled, the overlapping region is shown in the plots with a lighter color to indicate reduced reliability of the estimates.

**TABLE 1:** Total information capacities ($I_{\mathrm{tot}}$, bits) for the six sensor arrays used in the sensitivity and correlation map analyses.

Region	SQUID 102	SQUID 275	OPM 102	OPM 275	Triaxial OPM 102	Triaxial OPM 275
Cerebellum	216	247	254/223	328/284	458/397	544/464
Cortex	574	842	716/657	1588/1429	1625/1455	2997/2576
Whole brain	606	919	754/695	1647/1487	1766/1588	3246/2795

*Note:* For OPM arrays, the two values correspond to noise levels of 10 and $15\;\text{fT}/\sqrt{\mathrm{Hz}}$.

In the cerebellum, Triaxial OPM 275 achieved the highest information capacities, exceeding SQUID 102 by more than a factor of two at both noise levels. In the cortex, Triaxial OPM 275 provided again the largest gain, reaching nearly five times the capacity of SQUID 102 even with the higher noise level. Whole‐brain $I_{\mathrm{tot}}$ followed the same trend, increasing with both sensor count and the use of triaxial OPMs. Across all brain regions, raising the OPM noise level from 10 to $15\;\text{fT}/\sqrt{\mathrm{Hz}}$ reduced $I_{\mathrm{tot}}$ by approximately 10%–15%.

**FIGURE 6:**
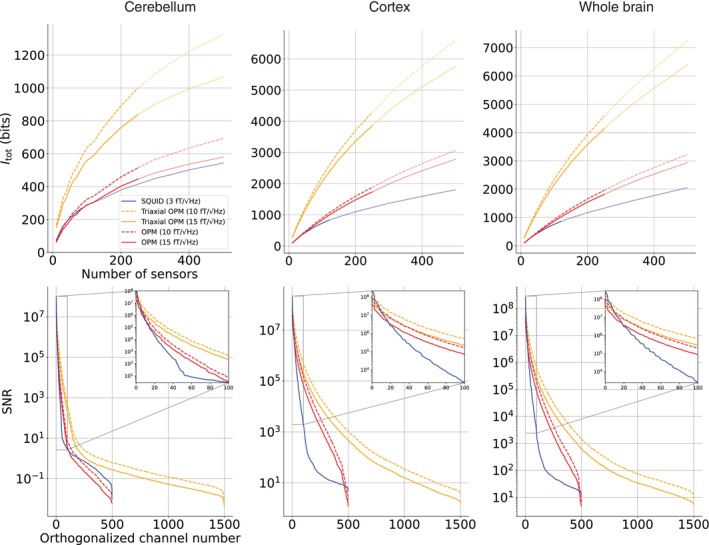
Total information capacity ($I_{\mathrm{tot}}$) for cerebellum, cortex, and whole brain as a function of sensor count. SQUID arrays are in blue, single‐axis OPMs in red, and triaxial OPMs in orange. Solid and dashed lines indicate OPM noise levels of 15 and $10\;\text{fT}/\sqrt{\mathrm{Hz}}$, respectively. SQUID noise level is fixed at $3\;\text{fT}/\sqrt{\mathrm{Hz}}$. The lighter‐colored plot segments denote the range where the average distance between neighboring sensor elements becomes smaller than the physical sensor length (28 mm for SQUIDs and 17 mm for OPMs). The bottom row shows the SNRs of the orthogonalized channels for the 500‐sensor arrays, with accompanying zoomed insets for the first 100 channels.

$I_{\mathrm{tot}}$ values for the increasing number of OPM and SQUID sensors are shown in Figure 6, with separate plots for the cerebellum, cortex, and whole brain. For OPMs, we again considered two noise levels: $10\;\text{fT}/\sqrt{\mathrm{Hz}}$ (dashed lines) and $15\;\text{fT}/\sqrt{\mathrm{Hz}}$ (solid lines). For SQUIDs, the noise level was fixed at $3\;\text{fT}/\sqrt{\mathrm{Hz}}$.

In the cerebellum, the SQUID arrays reach 544 bits at 500 sensors. OPMs achieve higher capacities, with 693 bits ($10\;\text{fT}/\sqrt{\mathrm{Hz}}$) and 579 bits ($15\;\text{fT}/\sqrt{\mathrm{Hz}}$) at 500 sensors. Triaxial OPMs approximately double these values, reaching 1323 and 1066 bits for the respective noise levels. While triaxial OPMs consistently outperform other modalities, single‐axis OPMs surpass SQUIDs only beyond approximately 120 sensors at the higher noise level and beyond about 50 sensors at the lower noise level.

In the cortex, information capacity continues to rise with sensor count. SQUIDs reach 1802 bits, while OPMs yield 3064 bits ($10\;\text{fT}/\sqrt{\mathrm{Hz}}$) and 2779 bits ($15\;\text{fT}/\sqrt{\mathrm{Hz}}$) at 500 sensors, clearly surpassing the SQUID performance. Triaxial OPMs again show a large advantage, with highest information values of 6588 and 5757 bits.

For the whole brain, the $I_{\mathrm{tot}}$ curves resemble those of the cortex but are consistently higher. SQUIDs reach 2051 bits, while OPMs yield 3223 and 2936 bits for the two noise levels. Triaxial OPMs achieve the highest capacities, with largest values of 7251 and 6396 bits. Importantly, the whole‐brain $I_{\mathrm{tot}}$ is not a simple sum of cortical and cerebellar values, reflecting overlap in the information captured from these regions.

Below the $I_{\mathrm{tot}}$ plots in Figure 6, we show the SNR values of the orthogonal channels for the 500‐sensor arrays, with separate plots for the cerebellum, cortex, and whole brain. While the $I_{\mathrm{tot}}$ analysis was based on sensors, these SNR values are based on channels, meaning that a 500‐sensor triaxial OPM array corresponds to 1500 channels.

In all regions, SQUIDs exhibit the lowest SNRs per channel, except for the first 20 channels. Single‐axis OPMs show higher SNRs overall, particularly, for the $10\;\text{fT}/\sqrt{\mathrm{Hz}}$ noise level, while triaxial OPMs further improve the SNR distribution. Across all modalities, SNR values decrease gradually with increasing channel number, but the decline is steeper for SQUIDs than for OPMs, as shown in the insets. Interestingly, in the cortex and whole‐brain plots, the single‐axis OPMs with the lower noise level and the triaxial OPMs with the higher noise level perform nearly on par for the first 60 channels, after which the triaxial OPMs maintain higher SNRs. This behaviour is also evident in the $I_{\mathrm{tot}}$ plots, where the corresponding curves closely follow each other at low sensor counts. However, for the cerebellum, triaxial OPMs maintain substantially higher SNRs already with low number of channels.

## 4 Discussion

### 4.1 Comparison of Sensor Types

We used forward modeling to compare OPM‐ and SQUID‐based MEG sensor arrays in detecting magnetic fields originating from the cerebellum. We included commercial SQUID‐sensor layouts from the Vectorview and CTF systems (102 magnetometers and 275 axial gradiometers; denoted as SQUID 102 and 275, respectively), as well as matched arrays of single‐axis (OPM 102 and 275) and triaxial OPMs (Triaxial OPM 102 and 275). We assessed their performance using sensitivity maps, subspace correlations of their cerebellar and cortical lead‐field matrices, and total information capacity $I_{\mathrm{tot}}$.

Overall, the sensitivity maps showed a clear benefit for OPMs over SQUIDs in detecting cerebellar activity. Compared to SQUID 102, single‐axis OPMs produced more than twice higher signal amplitudes in superficial cerebellar areas for the 102‐sensor configuration and over three times higher for the 275‐sensor configuration. Triaxial OPMs further increased the signal, reaching almost four times higher amplitudes with 102 sensors and over five times higher with 275 sensors. These improvements arise from the reduced sensor‐to‐scalp distance and the ability of triaxial sensors to capture the whole magnetic field vector at once. The deep cerebellar sources also produced stronger signals with both OPM types than with SQUIDs, which is reasonable given that the cerebellar mean signal strength was below 2 pT for both SQUID configurations, whereas all OPM configurations showed mean values starting from about 4 pT (OPM 102) and extending to almost 9 pT (Triaxial OPM 275), indicating better coverage of posterior cerebellar regions. However, in general, the deep cortical sources showed less improvement than the superficial sources, which reflects the general difficulty of MEG in accessing signals from the deep cortical areas.

When we scaled the sensitivity maps by the number of sensors in each array, Triaxial OPM 275 did not show the strongest sensitivity per sensor, but Triaxial OPM 102 provided the highest per‐sensor signal strength. This shows that the relationship between signal strength and sensor count is not linear. Adding sensors increases the total measured signal, but the marginal gain per sensor decreases, likely due to spatial oversampling. Beyond a certain sensor density, additional sensors may not substantially improve coverage or sensitivity unless they capture genuinely new and independent field information. However, having more sensors than the theoretical minimum is still advantageous, as it increases robustness against potential sensor failures. When comparing single‐axis and triaxial OPM arrays with the same number of sensors (but triaxial OPM array having three times more channels due to all orthogonal field components being measured), the sensitivity per sensor was higher in the triaxial arrays. This shows that measuring the full field vector is beneficial especially in the regime of medium‐density spatial sampling (102 sensors), but improvements were also seen in the regime of dense spatial sampling (275 sensors). This result originates from the fact that normal and the tangential sensors have different lead fields (Iivanainen et al. 2017): normal sensors are sensitive to the sources around the sensors, while tangential sensors are sensitive to sources directly beneath the sensor. Similar results have been shown by (Boto et al. 2022), where the authors show that triaxial sensors improve the coverage of the cortex.

The sensitivity maps also highlight a well‐known characteristic of MEG: signals that originate from gyri tend to be weaker than those from sulci as the former are parallel to the surface normals of the head‐conductivity boundaries (radial direction in the spherical conductor model). In this respect, the cerebellum is no different from the cortex. However, this orientation dependence is pronounced in the cerebellum, where small changes in the source orientation due to the cerebellum's high folding strongly affect measured signal strength. This makes the sensitivity maps patchy or spotted within the cerebellum.

The correlation maps provided complementary insight by showing that OPMs reduced lead field similarity between cerebellar and cortical sources. This reduction is important for separating signals originating from the cortex and the cerebellum. With OPM 102, the improvements relative to SQUID 102 were modest, but with denser layouts, as in the SQUID 275‐matched array (OPM 275), the reduction became more pronounced. Results for SQUID 102 and 275 were similar, showing that adding SQUID sensors beyond 100 sensors does not really benefit in this respect. Triaxial OPMs performed best, lowering correlation values well below 0.5 for most cortical sources. While OPM 275 already achieved strong field separability, the Triaxial OPM 102 reached similar results, demonstrating the benefit of triaxial sensors over single‐axis ones. With 275 OPMs, triaxial sensors only marginally reduce the correlations compared to single‐axis ones. This result shows that close‐to‐minimal lead‐field correlations can be achieved with 102 triaxial OPMs. In contrast, OPM 102 without triaxial channels still left many cortical areas critical for cerebellar investigations highly correlated. When we summarized the correlation results by the percentage of highly correlated subspace angles, the overall trends followed the full correlation maps. Even with OPM 102, the mean percentage of highly correlated subspaces was reduced to 50%.

The correlation maps show that the correlations between the cerebellum and the cortex decrease as a function of their distance, as expected. The highest cortico‐cerebral lead‐field correlations, regardless of the sensor array, are in the posterior cortical regions and in the medial occipital cortex. With OPM 275 and Triaxial OPM 102, the posterior regions were less influenced, but the medial regions remained highly correlated. For example, the fusiform gyrus showed little benefit from increasing the number of sensors and only a small improvement when moving from SQUIDs to OPMs. Altogether, the correlation analysis indicates that the OPM arrays could reduce the spatial leakage of the cerebellar source estimates to most source regions, for example, to the primary visual cortex.

Total information capacity $I_{\mathrm{tot}}$ further supported the advantage of OPMs, particularly of triaxial sensors. Across the whole brain, OPMs outperformed SQUIDs even with similar channel counts. For the cerebellum, triaxial OPMs provided a clear advantage, while single‐axis OPMs exceeded SQUID performance with sensor counts above 50 and 120 for lower and higher noise levels, respectively. This suggests that for cerebellar studies using single‐axis OPMs with around 100 sensors, achieving meaningful information gains requires lower noise levels. With triaxial OPMs, the benefits were already visible at lower numbers of sensors and higher noise levels. Although a 500‐sensor whole‐head array is not yet realistic, local high‐density triaxial arrays of about 100 sensors could already provide substantial improvements. The increased information capacity reflects both closer proximity to sources and improved spatial sampling of OPMs over SQUIDs, even when they have a higher noise level (10–15 vs. 3 fT/$\sqrt{\mathrm{Hz}}$).

SNR as a function of the orthogonal channels in the 500‐sensor arrays also demonstrate the benefits of triaxial sensors in measuring the cerebellum. Triaxial OPMs maintained higher SNR values across the orthogonal channels of the entire 500‐sensor array, while single‐axis OPMs still competed with SQUIDs in the cerebellum. The SNR plots also showed differences between the cortex and the cerebellum. In the cortex and the whole brain, single‐axis OPMs with 10 fT/$\sqrt{\mathrm{Hz}}$ noise level performed nearly on par with triaxial OPMs with 15 fT/$\sqrt{\mathrm{Hz}}$ noise for the first 60 orthogonal channels. In contrast, in the cerebellum, triaxial OPMs with 15 fT/$\sqrt{\mathrm{Hz}}$ noise showed higher SNR than single‐axis sensors with 10 fT/$\sqrt{\mathrm{Hz}}$ noise.

We also evaluated the conservation factor C=A/B, which quantifies the relationship between the net signal produced by simultaneously active sources ($A=∥\sum_{j}L_{:,j}{∥}_{2}$) and the absolute signal defined as the sum of the signals produced if these sources were activated individually ($B=\sum_{j}∥L_{:,j}{∥}_{2}$). Consistent with earlier studies (Ahlfors et al. 2010; Samuelsson, Sundaram, et al. 2020), C did not differ across the sensor types and arrays. This likely reflects the dominance of low spatial frequency components in MEG signals, which are more sensitive to signal cancellation due to nearby sources. Even though OPMs are closer to the scalp, the basic nature of MEG measurements limits improvements in C. Moreover, this metric does not include sensor or external noise, so the increased SNR from reduced sensor‐to‐brain distance does not directly affect C. Together with previous findings, our results confirm that while OPMs improve sensitivity and reduce spatial correlation of field patterns, they do not alter the overall cancellation pattern.

Motivated by the work by Iivanainen et al. (2017), where the authors suggested that tangential OPM sensors may be more sensitive to forward‐model errors than normal‐component measuring OPMs and SQUIDs, we briefly assessed the sensitivity of the OPM and SQUID arrays to forward‐model errors in the cortex and the cerebellum. We compared cortical and cerebellar topographies computed with three‐layer and one‐layer BEM models by computing the relative error (RE) and correlation coefficient (CC) between them (Iivanainen et al. 2017). For the cortical topographies, the mean RE (CC) was 17.79% (0.98), 18.39% (0.98), and 22.10% (0.97) for normal‐component OPMs, tangential OPMs, and SQUIDs, respectively. The corresponding mean REs (CCs) for the cerebellar sources were 24.29% (0.97), 24.11% (0.97), and 28.05% (0.96), respectively. This analysis suggests that tangential OPMs are not necessarily more sensitive to forward‐modeling errors than normal‐component OPMs, while SQUIDs might be slightly more sensitive to those errors than OPMs. However, the analysis clearly suggests that cerebellar sources are affected more by the head model than cortical sources, indicating that more accurate forward modeling may be required for the cerebellum.

We acknowledge that more studies are needed to fully understand the head‐modeling requirements for the cerebellum and the cortex as well as for the radial and tangential OPMs. Especially, it would be of interest to assess the need of four‐layer BEM models (Stenroos and Nummenmaa 2016) or even more detailed approaches based on finite element models (Vorwerk et al. 2014) for accurate cerebellar source estimation. In future studies, numerical and anatomical errors in the head modeling should be analyzed separately as the sensor arrays might have a different sensitivity to them.

### 4.2 Practical Implications

The methods we introduced here provide practical tools for planning cerebellar MEG studies. Sensitivity maps can help identify promising target regions and show, for instance, that the flocculonodular lobe is more difficult to measure than posterior cerebellar lobes. They also underline the challenges of accessing deep cortical regions, where further advances in imaging methods are still needed. Correlation maps, in turn, can help evaluate potential ambiguities between cerebellar and cortical activity. For example, if we observe activation in a cortical area, we can compare its field correlation against candidate cerebellar regions to test whether the cerebellar and cortical activity can be reliably separated. This is especially useful when studying visual networks, as several cerebellar regions involved, particularly, around the posterior lobule VI, lie close to the ventral occipitotemporal cortex, making the distinction between cerebellar and occipital signals critical (Claeys et al. 2003).

### 4.3 Limitations

One limitation of this study concerns the cerebellar model resolution. Sensitivity maps were computed on a high‐resolution source space, allowing detailed spatial comparison. However, due to computational constraints, correlation maps and $I_{\mathrm{tot}}$ metrics were based on a lower‐resolution model. This may slightly reduce spatial precision and underestimate potential differences between sensor types.

Additionally, in $I_{\mathrm{tot}}$ simulations we did not take into account some aspects of real behaviour of sensors and sensor arrays. As seen in Figure 1B, the SQUID sensors started to overlap at high sensor counts. In realistic SQUID arrays, the sizes of the SQUID pick‐up loops would be reduced to prevent sensor overlap. This in turn increases the SQUID sensor noise, which would affect the $I_{\mathrm{tot}}$ calculations so that $I_{\mathrm{tot}}$ would plateau or even reduce at the point where the SQUID pick‐up loops start to overlap (Nenonen et al. 2004). Thereby, our $I_{\mathrm{tot}}$ curves overestimate the information capacity of SQUID sensor arrays at high sensor counts. This same also applies for OPMs (Bezsudnova et al. 2022), but to a lesser extent: OPMs are inherently smaller than SQUIDs and high‐density multichannel OPMs can be constructed by using large vapor cells containing multiple channels (see, e.g., Xu et al. 2025).

Another limitation in our $I_{\mathrm{tot}}$ calculations is related to the noise in single‐axis versus triaxial OPMs. Typically, triaxial OPMs have a higher noise floor than single‐axis ones. However, comparison between $I_{\mathrm{tot}}$ curves of triaxial OPMs with a noise floor of 15 fT/$\sqrt{\mathrm{Hz}}$ and single‐axis ones with a 10 fT/$\sqrt{\mathrm{Hz}}$ noise floor should give a realistic view as these noise figures are close to what is typically achieved with single‐axis and triaxial OPM sensors (QuSpin Inc. 2025).

### 4.4 Future Directions

We studied the utility of uniform whole‐head OPM‐based sensor arrays in detecting cerebellar activity. However, OPMs offer flexible sensor placement and, in principle, partial‐coverage sensor arrays that target the cerebellum could be designed and constructed using sensor‐array‐optimization methods (see, e.g., Iivanainen et al. 2021). Given the technical and financial constraints of whole‐head OPM arrays with many sensors, locally increased sensor density, especially around the cerebellum, may allow to reach similar performance with a reduced sensor count. In addition, it would be interesting to study the reach of the cerebellar magnetic fields to outline the area where sensor placing is beneficial.

Future studies could also include more detailed analyses of signal cancellation. For instance, computing the conservation factor C as a function of spatial frequency could reveal differences between systems not captured by the global metric alone.

Finally, these results point toward a broader shift in MEG research. Cerebellar activity has often been excluded by convention rather than necessity. Our findings show that the magnetic field patterns from cortical sources overlap with those from the cerebellum, underlining the importance of including the cerebellum in future MEG studies. Moving toward whole‐brain MEG source analysis will be essential for a more complete understanding of human brain dynamics.

## 5 Conclusion

We studied the use of OPMs over conventional SQUID sensors in detecting cerebellar activity. Our results indicate clear benefits for OPMs in terms of sensitivity, lead‐field correlations between the cortex and the cerebellum, as well as total information. Triaxial OPMs showed better performance than single‐axis OPMs in measuring the cerebellum. Importantly, our results indicate that the improvements provided by OPMs over SQUIDs can already be achieved with 102 triaxial OPM sensor arrays covering the whole head.

## Funding

This work was supported by the National Institute of Neurological Disorders and Stroke (R01NS104585).

## Conflicts of Interest

The authors declare no conflicts of interest.

## Data Availability

The data that support the findings of this study are available from the corresponding author upon reasonable request.
